# Decrease in Anti-HBs Antibodies over Time in Medical Students and Healthcare Workers after Hepatitis B Vaccination

**DOI:** 10.1155/2017/1327492

**Published:** 2017-09-26

**Authors:** H. V. Sahana, N. Sarala, S. R. Prasad

**Affiliations:** ^1^Department of Pharmacology, Sri Devaraj Urs Medical College, Sri Devaraj Urs Academy of Higher Education and Research, Tamaka, Kolar, Karnataka, India; ^2^Department of Microbiology, Sri Devaraj Urs Medical College, Sri Devaraj Urs Academy of Higher Education and Research, Tamaka, Kolar, Karnataka, India

## Abstract

**Background:**

Hepatitis B is one of the most important occupational hazards among healthcare workers (HCWs). This study aimed to measure the anti-HBs titres among the medical students and HCWs vaccinated against hepatitis B virus and to determine the association between anti-HBs levels and time since vaccination.

**Materials and Methods:**

In this cross-sectional study, medical students and healthcare workers who had received all three doses of hepatitis B vaccination and completed at least six months after vaccination since the last dose were included. 3 ml blood was collected from subjects (*n* = 340) and anti-HBs titre was estimated using ELISA.

**Results:**

A total of 340/400 subjects were aged between 18 and 60 years; 204 were females and 136 males. The median and interquartile range for time since vaccination were 5 and 5 years, respectively. Duration since vaccination was ≤5 years in 223 (65.5%), 6–10 years in 84 (24.7%), and >10 years in 33 (9.70%); among them, antibody titres were >10 mIU/ml in 94.1%, 79.7%, and 72.7% subjects, respectively. There was significant decline in antibody titres as duration of postvaccination increased.

**Conclusion:**

The proportion of subjects who were unprotected after 5 and 10 years after vaccination were 20% and 27%, respectively. The need for a booster dose can be made mandatory at least for healthcare professionals.

## 1. Introduction

Hepatitis B virus (HBV) infection is a blood-borne disease affecting around 2 billion people worldwide of which about 350 million develop chronic hepatitis infection leading to complications like chronic active hepatitis, cirrhosis of liver, and hepatocellular carcinoma [1]. Hepatitis B infection is one of the most important occupational hazards among medical students and healthcare workers (HCWs). HBV is highly contagious which gets transmitted by exposure to infected blood or body fluids and by injuries with contaminated sharp objects like needles. Due to frequent handling of blood and body fluids of patients, HCWs are four times more at risk of contracting hepatitis B infection compared to the general population [2]. The risk of acquiring this infection among the nonvaccinated individuals ranges within 6–30% following single exposure. According to WHO, 5.9% of HCWs are exposed annually to blood-borne HBV infections which correspond to about 66,000 worldwide [3].

Vaccination is the effective means of prevention of HBV infection. Hepatitis B vaccine is available since 1982 which was initially derived from plasma and after 1984 it is available as recombinant vaccine [4]. In 1997, Centre for Disease Control and Prevention (US CDC) has recommended that all HCWs should receive a complete course of hepatitis B vaccination at 0, 1, and 6 months which is administered intramuscularly [5]. A review emphasizes the need to educate the healthcare workers about hepatitis B infection, available vaccines, postvaccine immune status, and postexposure prophylaxis [6].

Testing for evidence of protective immunity to HBsAg vaccination is required as some individuals do not develop sufficient levels of antibodies against HBsAg (anti-HBs). An anti-HBs titre less than 10 mIU/ml is regarded as nonresponse, levels between 10 and 100 mIU/ml are considered as hyporesponse and more than 100 mIU/ml is considered as high level of immunity following vaccination. Levels more than 10 mIU/ml at any time after vaccination are considered as a marker of sustained immunity which provides protection against infection [7].

A study has shown that six (10.5%) of all successfully vaccinated persons had not attained minimal protective levels of antibody of 10 mIU/ml, six (10.5%) had antibody levels in the range of >11–100 mIU/ml, and 45 (79%) had antibody levels >101 mIU/ml [8]. Another study conducted on 112 HCWs has shown that protective antibody levels were 99.9% one year after vaccination and decreased to 80.96% and 46.16% after 5 and 10 years after vaccination, respectively [9]. A North Indian study among 166 HCWs has reported that the anti-HBs titre <10 mIU/ml was more common in participants whose vaccination was >5 years (36.8%) as compared to those <5 years (24.4%), which was significant (*p* = 0.04) [10]. Age, gender, obesity, smoking, immunity, and genetic factors may be responsible for reduced immune response to vaccination [11, 12]. This study was undertaken to evaluate the immune response among the medical students and healthcare workers in our hospital after various durations of hepatitis B vaccination.

## 2. Materials and Methods

**Figure 1:**
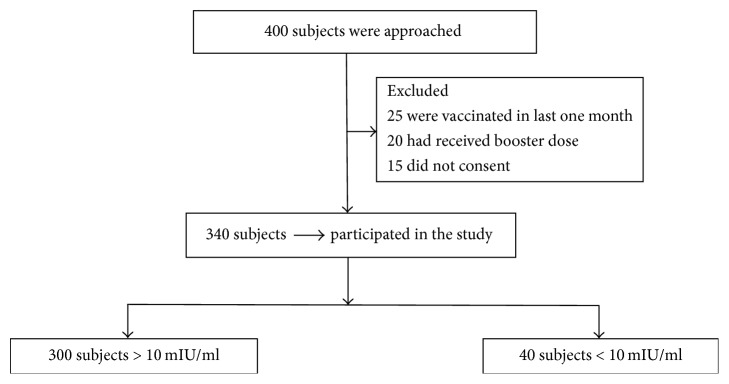
Screening, recruitment of subjects, and antibody titre.

A cross-sectional study was conducted by the Departments of Pharmacology and Microbiology at R. L. Jalappa Hospital and Research Centre attached to Sri Devaraj Urs Medical College, Kolar, for a period of five months from May to September 2015. The protocol was approved by Institutional Ethics Committee. Written informed consent was obtained from all the subjects. Doctors, nurses, medical students, interns, postgraduates, nursing students, technicians, and housekeeping staffs who had received all 3 doses of hepatitis B vaccination and completed at least six months of postvaccination period were included (Figure 1). The present study represents 20% of medical students and 57% of HCWs; HBV vaccination is covered by the institute. Exclusion criteria were those who had received booster dose in the last 5 years and history of hepatitis B infection, chronic liver disease, and diabetes mellitus and those who were on prolonged steroid therapy. The confounding factors like age, gender, and BMI were assessed using Pearson correlation followed by multiple stepwise logistic regression. History of smoking and alcohol consumption was revealed by only 3% and it was sensitive issue to be elicited in our setting and evaluation of genetic variation, immune status, chronic subclinical infections were not done due to logistic difficulties. A proforma was given to all subjects to capture the demographic details like age, gender, occupation, body mass index (BMI), and hepatitis B vaccination status.

Under strict aseptic precautions, 3 ml of venous blood was collected from all eligible subjects in a vacutainer containing clot activator (silicone and micronized silica particles manufactured by BD, Franklin Lakes, NJ, USA). Serum separation was performed by centrifugation of the blood sample at 3000 rpm for 5 minutes at room temperature. Serum thus separated was stored at −80°C until further analysis. The quantification of serum anti-HBs level was done by Enzyme Linked Immuno Sorbent Assay technique using a commercially available kit (DSI ELISA, Italy) strictly adhering to the manufacturer's protocol.

## 3. Statistical Methods

Descriptive and inferential statistics were used to analyse the data. The quantitative variables age, BMI, and time since vaccination were expressed as mean and standard deviation while categorical variables, namely, gender and occupation, were expressed as percentage. ANOVA and post hoc Bonferroni were done for antibody titres between different age groups. Odds ratio was used for calculation of protective antibody titre. Association between antibody titres and time since vaccination, age, gender, and BMI were analysed using Pearson correlation. Multiple stepwise logistic regression was used to identify the factors influencing antibody titre levels. Statistical Package for Social Sciences (SPSS) Version 22.0, manufactured by International Business Management (IBM) Corporation, was used for statistical analysis. *p* < 0.05 was considered statistically significant.

## 4. Results

There were 340 subjects of which 204 (60%) were females and 136 (40%) males, and the age of subjects ranged from 18 to 60 years. Forty-nine percent of them were in the age group of 18–24 years and only 3.5% were more than 45 years (Table 1).

**Figure 2:**
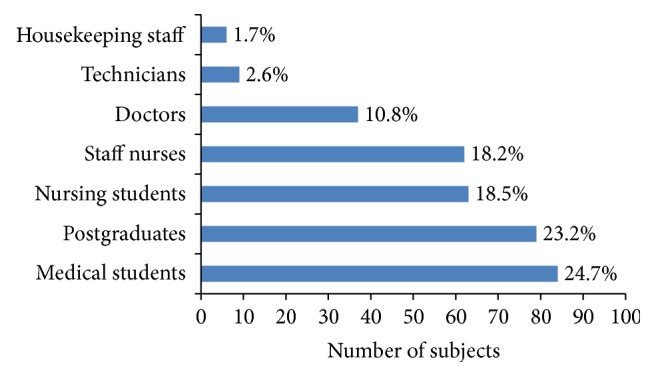
Percentage of vaccinated subjects.

Vaccinated HCWs of different categories are represented in Figure 2. The mean time since vaccination for all the subjects was 5.4 ± 3.8 years. The mean antibody titres in relation to postvaccination time are depicted in Table 2 and it reduced significantly (*p* = 0.0001) as the time since vaccination increased. The protective levels of antibody persisted in 94.1%, 79.7%, and 72.7% of subjects as shown in Table 2. The subjects with duration of vaccination ≤ 5 years were 4.2 times protected compared to those with more than 5 years which was calculated using odds ratio.

**Figure 3:**
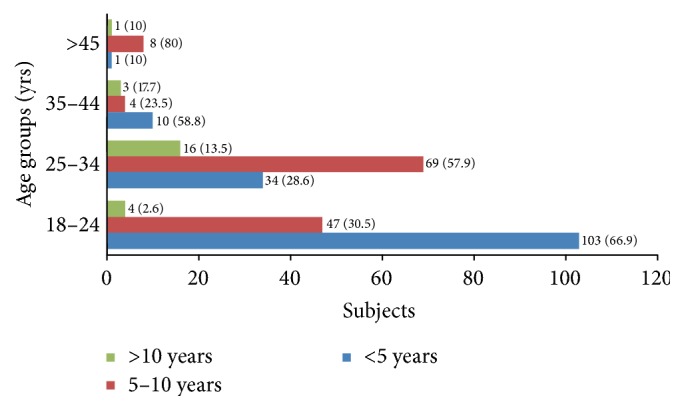
Subjects with anti-HBs titres more than 10 mIU/ml and postvaccination duration. Percentage is represented in parentheses.

Among the three hundred subjects with anti-HBs titres, more than 10 mIU/ml in different age groups and variable period of postvaccination are 154 (51.3%), 119 (39.6%), 17 (5.6%), and 10 (3.3%) as depicted in Figure 3.

**Figure 4:**
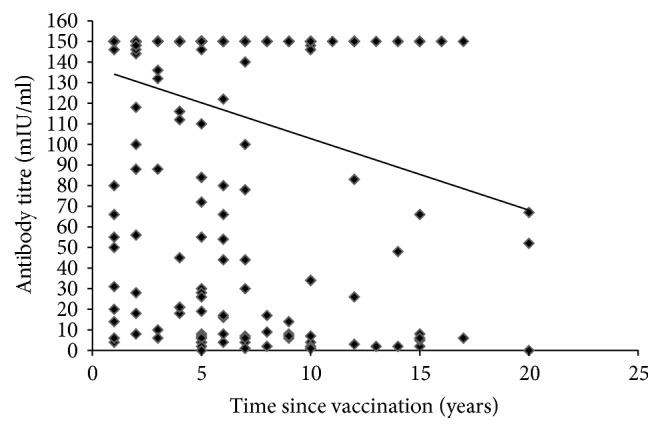
Correlation between anti-HBs levels and duration.

Subjects with titres more than 10 mIU/ml were 276 when duration of vaccination was less than 10 years. 248/340 subjects had antibody titres above 135 mIU/ml, which are overlapping, and 92 of them had titres below 134 mIU/ml. Pearson correlation showed a negative linear relationship between the anti-HBs titres and time since vaccination which was statistically significant (*r* = −0.24, *p* = 0.001) (Figure 4).

There was no significant association between decline in antibody levels with age (*p* = 0.242), gender (*p* = 0.108), and BMI (*p* = 0.516) of the subjects. There were 44 subjects who had received complete course of vaccination in the last one year of which 34 (82.92%) had high level of immune response with anti-HBs > 100 mIU/ml, 7 (17.07%) were hyporesponders with titre between 10 and 100 mIU/ml, and 3 were nonresponders with titre < 10 mIU/ml.

## 5. Discussion

Hepatitis B infection is a major cause of liver cirrhosis and hepatocellular carcinoma. Vaccination is the effective way of preventing the infection and its complications. Anti-HBs is an important serological marker to assess vaccine induced immunity to HBV. In developing countries, 40–65% of HBV infections in healthcare workers have been attributed to percutaneous occupational exposure in contrast to developed countries, where it is less than 10%, largely because of immunization and postexposure prophylaxis [13]. Hence healthcare workers need to be vaccinated due to the increased risk of occupational exposure. In our study, we have analysed the data of 340 vaccinated subjects which represents 57% of HCWs, and two studies in Delhi have reported 52–59% and 55.4% [6, 14].

Majority were in the age range of 18–24 years and 60% were females which is similar to study by Rao et al., whereas two other studies showed male predominance [8–10]. The overall seroprotection rate was 88.2% irrespective of the duration of vaccination and in other studies it was 89% and 70% with sample size of 57 and 166, respectively [8, 10]. There was a significant decline in antibody titres as the years of vaccination prolonged. In the present study, 80% subjects were protected 6–10 years after vaccination and 72% after 10 years, as compared to study by Mahawal et al. which observed 80.9%, 5 years after vaccination, and further decreased to 46.1%, after 10 years [9]. In our study, the odds of seroprotection were 4.2 times greater in individuals when duration of vaccination was less than or equal to 5 years.

Pearson correlation indicates negative association of the anti-HBs titres as the time progressed. When time since vaccination was considered, the protection rate declined and there was statistically significant reduction in anti-HBs titres beyond 10 years of postvaccination. Our study is comparable with previous study conducted in Iran by Norouzirad et al., which showed seroprotection rate of 65% at five years after vaccination with significant decline over time [15]. Similar study conducted by Jafarzadeh and Montazerifar, among healthy Iranian children which evaluated persistence of protective antibody level after 10 years of primary vaccination, reported that only 47.9% of children had protective level of HBsAb > 10 mIU/ml which is low compared to our study [16]. We studied the persistence of protective level of antibody in individuals at different time period of postvaccination and it was 94%, 80%, and 72% as years progressed as depicted in Table 2, and another study has reported 99.9%, 80.96%, and 46% [9]; this helps us to find out their immune status.

Previous studies have shown that age of the individual influences the immune response to vaccination, seroconversion is slow, and seroprotection is less among individuals above 40 years compared to those less than 40 years [17, 18]. However, we did not find any association between age of the subject and the rate of seroprotection probably because majority of our subjects were less than 40 years. Though females formed the majority in our study, we did not observe gender difference with respect to immune response following vaccination. Previous studies have reported increased percentage of nonresponders among males compared to females [17, 19]. Smoking and genetic factors (certain HLA types) are other factors known to be associated with reduced immune response [11, 12, 20], but we could not assess them.

During the conduct of the study, we created awareness by providing information about the need for HBV vaccination among the hospital staff (housekeeping staff and laboratory technicians) who are at highest risk. The subjects were also informed about their antibody titres. All hospital staff are to be sensitized towards the need for receiving a complete course of hepatitis B vaccination and estimate anti-HBs titres periodically so that booster dose can be advised as and when required. Hospitals should implement policy to vaccinate all categories of HCWs at the time of recruitment followed by postvaccination measurement of antibody titres which is cost-effective measure compared to postexposure prophylaxis with immunoglobulin which is expensive. This practice has to be implemented as a healthcare measure in all hospitals.

Limitations of our study were that we were not able to evaluate the association of decreased immune response with risk factors like smoking, alcoholism, nutritional status, chronic infections, site of administration of vaccine, and genetic factors using Pearson correlation followed by multiple stepwise logistic regression, which also contribute to reduced immune response. Further, monitoring of vaccinated subjects periodically at an interval of 5 years after vaccination would help to find out their antibody titres and the need for booster dose can be emphasized.

## 6. Conclusion

Our findings reveal that there was a decline in antibody titres as the time since vaccination progressed. The proportion of patients who were unprotected after 5 and 10 years after vaccination were 20% and 27%, respectively. Majority had postvaccination protection against hepatitis B infection up to 10 years. Estimation of antibody titres after 5 and 10 years will determine the need for a booster dose which can be made mandatory at least for healthcare professionals.

## Figures and Tables

**Table 1:** Gender, age, and BMI of the study subjects.

Gender	Age range in years		18–24	25–34	35–44	>45	Total
Male	Number of subjects		**38**	**78**	**13**	**07**	**136**
	Age (years)	Mean ± SD	22.1 ± 1.5	27.0 ± 1.9	37.0 ± 1.7	53.7 ± 7.4	28.2 ± 7.6
		Median (IQR)	22.0 (2.0)	27.0 (2.2)	38.0 (3.5)	52.0 (14.0)	27.0 (5.0)
	BMI	Mean ± SD	23.0 ± 3.2	24.2 ± 3.1	26.3 ± 5.0	24.9 ± 3.9	24.3 ± 3.4
		Median (IQR)	24.4 (3.4)	23.7 (3.7)	25.4 (7.1)	26.5 (7.4)	23.9 (4.3)

Female	Number of subjects		**130**	**60**	**09**	**05**	**204**
	Age (years)	Mean ± SD	21.3 ± 1.7	27.7 ± 2.5	38.3 ± 2.9	51.2 ± 5.5	24.7 ± 6.3
		Median (IQR)	21.0 (3.0)	27.0 (3.0)	39.0 (5.0)	49.0 (10.5)	23.0 (5.0)
	BMI	Mean ± SD	21.3 ± 3.9	22.9 ± 3.9	23.7 ± 3.2	25.2 ± 2.3	22.0 ± 3.9
		Median (IQR)	21.0 (4.2)	22.3 (5.1)	24.4 (5.6)	26.6 (4.2)	21.5 (4.7)

Total *n* (%)			168 (49.4)	138 (40.5)	22 (6.4)	12 (3.5)	340

**Table 2:** Subjects with duration of vaccination and antibody titres.

Time since vaccination (years)	Number of subjects (%)	Subjects protected (>10 mIU/ml) *n* (%)	Subjects not protected (<10 mIU/ml) *n* (%)	Antibody titre Mean ± SD	*p* value ANOVA
≤5	223 (65.6)	209 (94.1)	14 (5.8)	128.4 ± 46.2	0.0001
6–10	84 (24.7)	67 (79.7)	17 (20.2)	102.5 ± 63.4^*∗*^	0.0001
>10	33 (9.7)	24 (72.7)	9 (27.2)	93.2 ± 66.1^*∗*^	0.0001
Total	340	300	40	118.6 ± 54.6	

*p* = 0.0001 between the groups (ANOVA); ^**∗**^*p* = 0.001 ≤ 5 versus 6–10 years and ≤5 versus >10 years (Bonferroni); *p* = 1.000 6–10 versus >10 years.

## References

[1] Chan J., Burns M. A., Wells B. G., Schwinghammer T. L., Malone P. M., Kolesar J. M., Dipiro J. T. (2013). Viral hepatitis. *Pharmacotherapy Principles and Practice*.

[2] Ciorlia L. A. S., Zanetta D. M. T. (2005). Hepatitis B in healthcare workers: prevalence, vaccination and relation to occupational factors. *Brazilian Journal of Infectious Diseases*.

[3] Pruss-Ustün A., Rapiti E., Hutin Y. (2005). Estimation of the global burden of disease attributable to contaminated sharps injuries among health-care workers. *The American Journal of Industrial Medicine*.

[4] Mast E., Mahoney F., Kane M., Margolis H., Plotkin S. A. (2004). Hepatitis B vaccine. *Vaccines*.

[5] Immunization of Health Care Workers (1997). Recommendation of advisory committee on immunization practice (ACIP) and the Hospital Infection Control Practice advisory committee (HICPAC). *MMWR, Recommendation and Report*.

[6] Singhal V., Bora D., Singh S. (2009). Hepatitis B in health care workers: Indian scenario. *Journal of Laboratory Physicians*.

[7] Chathuranga L. S., Noordeen F., Abeykoon A. M. S. B. (2013). Immune response to hepatitis B vaccine in a group of health care workers in Sri Lanka. *International Journal of Infectious Diseases*.

[8] Rao T. V., Suseela I. J., Sathiavathy K. A. (2008). Estimation of antibodies to HBsAg in vaccinated health care workers. *Indian Journal of Medical Microbiology*.

[9] Mahawal B. S., Bhai N., Kataria V. K., Gulati N., Chandola I. (2013). Estimation of Anti Hbs antibody titer in adults during 5–10 years period following three doses of vaccine. *IOSR Journal of Pharmacy and Biological Sciences*.

[10] Batra V., Goswami A., Dadhich S., Kothari D., Bhargava N. (2015). Hepatitis B immunization in healthcare workers. *Annals of Gastroenterology*.

[11] Tripathy S., Sathi H. C., Puspa Saha S., Shankar R., Singh V. K. (2011). Study of immune response after hepatitis B vaccination in medical students and healthcare workers. *Indian Journal of Preventive and Social Medicine*.

[12] Nashibi R., Alavi S. M., Yousefi F. (2015). Post-vaccination immunity against hepatitis B virus and predictors for non-responders among medical staff. *Jundishapur Journal of Microbiology*.

[13] Hutin Y., Hauri A., Chiarello L. (2003). Best infection control practices for intradermal, subcutaneous, and intramuscular needle injections. *Bulletin of the World Health Organization*.

[14] Sukriti, Pati N. T., Sethi A. (2008). Low levels of awareness, vaccine coverage, and the need for boosters among health care workers in tertiary care hospitals in India. *Journal of Gastroenterology and Hepatology (Australia)*.

[15] Norouzirad R., Shakurnia A. H., Assarehzadegan M.-A., Serajian A., Khabazkhoob M. (2014). Serum levels of anti-hepatitis B surface antibody among vaccinated population aged 1 to 18 years in Ahvaz city Southwest of Iran. *Hepatitis Monthly*.

[16] Jafarzadeh A., Montazerifar S. J. (2006). Persistence of anti-HBs antibody and immunological memory in children vaccinated with hepatitis B vaccine at birth. *Journal of Ayub Medical College, Abbottabad*.

[17] Zeeshan M., Jabeen K., Ali A. N. (2007). Evaluation of immune response to Hepatitis B vaccine in health care workers at a tertiary care hospital in Pakistan: an observational prospective study. *BMC Infectious Diseases*.

[18] Lim W. L., Wong D. A., Cheng K. C. (1996). Immune response to hepatitis B vaccine in health care workers in Hong Kong. *Hong Kong Medical Journal*.

[19] Wood R. C., Macdonald K. L., White K. E., Hedberg C. W., Hanson M., Osterholm M. T. (1993). Risk factors for lack of detectable antibody following hepatitis B vaccination of minnesota health care workers. *The Journal of the American Medical Association*.

[20] Das K., Gupta R. K., Kumar V., Kar P. (2003). Immunogenicity and reactogenicity of a recombinant hepatitis B vaccine in subjects over age of forty years and response of a booster dose among nonresponders. *World Journal of Gastroenterology*.

